# The Bones of Children With Obesity

**DOI:** 10.3389/fendo.2020.00200

**Published:** 2020-04-24

**Authors:** Danilo Fintini, Stefano Cianfarani, Marta Cofini, Angela Andreoletti, Grazia Maria Ubertini, Marco Cappa, Melania Manco

**Affiliations:** ^1^Endocrinology Unit, Pediatric University Department, Bambino Gesù Children's Hospital, Rome, Italy; ^2^Diabetes and Growth Disorders Unit, Dipartimento Pediatrico Universitario Ospedaliero Bambino Gesù Children's Hospital, Tor Vergata University, Rome, Italy; ^3^Department of Women's and Children's Health, Karolinska Institute and University Hospital, Stockholm, Sweden; ^4^Pediatric Clinic, Department of Surgical and Biomedical Sciences, University of Perugia, Perugia, Italy; ^5^Pediatric Resident, Pediatric Clinic, University of Brescia, Brescia, Italy; ^6^Research Area for Multifactorial Diseases, Bambino Gesù Children's Hospital, Rome, Italy

**Keywords:** bone, child, inflammation, lifestyle, obesity, physical activity, polyphenols, polyunsaturated fatty acids

## Abstract

Excess adiposity in childhood may affect bone development, ultimately leading to bone frailty. Previous reports showing an increased rate of extremity fractures in children with obesity support this fear. On the other hand, there is also evidence suggesting that bone mineral content is higher in obese children than in normal weight peers. Both adipocytes and osteoblasts derive from multipotent mesenchymal stem cells (MSCs) and obesity drives the differentiation of MSCs toward adipocytes at the expense of osteoblast differentiation. Furthermore, adipocytes in bone marrow microenvironment release a number of pro-inflammatory and immunomodulatory molecules that up-regulate formation and activation of osteoclasts, thus favoring bone frailty. On the other hand, body adiposity represents a mechanical load, which is beneficial for bone accrual. In this frame, bone quality, and structure result from the balance of inflammatory and mechanical stimuli. Diet, physical activity and the hormonal milieu at puberty play a pivotal role on this balance. In this review, we will address the question whether the bone of obese children and adolescents is unhealthy in comparison with normal-weight peers and discuss mechanisms underlying the differences in bone quality and structure. We anticipate that many biases and confounders affect the clinical studies conducted so far and preclude us from achieving robust conclusions. Sample-size, lack of adequate controls, heterogeneity of study designs are the major drawbacks of the existing reports. Due to the increased body size of children with obesity, dual energy absorptiometry might overestimate bone mineral density in these individuals. Magnetic resonance imaging, peripheral quantitative CT (pQCT) scanning and high-resolution pQCT are promising techniques for the accurate estimate of bone mineral content in obese children. Moreover, no longitudinal study on the risk of incident osteoporosis in early adulthood of children and adolescents with obesity is available. Finally, we will address emerging dietary issues (i.e., the likely benefits for the bone health of polyunsaturated fatty acids and polyphenols) since an healthy diet (i.e., the Mediterranean diet) with balanced intake of certain nutrients associated with physical activity remain the cornerstones for achieving an adequate bone accrual in young individuals regardless of their adiposity degree.

## Introduction

The public health burden of epidemic obesity in childhood has been increasing worldwide in the last three decades. When childhood obesity persists to adulthood, the risk of developing chronic diseases early in life is significantly increased (1). The last report of the World Health Organization (WHO) (2) shows that about 800,000 children in the WHO European Region suffer from severe obesity. According to Global Health Observatory data, 18% of youth aged 5–19 years-old worldwide were overweight or obese in 2016 (3, 4).

Growing evidence suggests that adiposity influences the child bone health. Previous studies found that children and adolescents with obesity have bone mineral content (BMC) (see glossary in Table 1) higher than normal-weight peers, indicating that the adipose tissue exerts a positive effect on bone structure (5–7). On the other hand, it has been reported an increased rate of extremity fractures in children with obesity, suggesting poorer bone quality (8, 9).

**Table 1:** Glossary of radiological bone parameters.

**Bone parameters**	**Unit**	**Definition**
Bone Mineral Content (BMC)	G	Sum of all skeletal tissue within the body measured by the densitometer
Areal Bone Mineral Density (aBMD)	g/cm^2^	Mineral mass of bone per unit area of the two-dimensional projection image
Bone mineral apparent density (BMAD)	g/cm^3^	Volumetric density (derived from the formula BMD/√ bone area); less dependent on bone size and body stature
Volumetric Bone Mineral Density (vBMD)	g/cm^3^	Cortical or trabecular density

In young people with obesity, bone quality and structure result from the balanced effects of enhanced release of inflammatory and immunomodulatory cytokines (10, 11) and mechanical overload. Interestingly, both the adipose tissue and the bone are metabolically active organs, due to the constant production and release of molecules, cytokines and hormones. These molecules modulate in an endocrine and paracrine fashion a number of metabolic activities, whole body inflammatory status and energy metabolism (12, 13). Immune cells embedded in the adipose tissue contribute to the interplay between adipose tissue and bone, while mechanical stimuli exerted by the adipose tissue on the bone structure generate and/or amplify molecular signals. There is evidence suggesting a cross-talk between adipose tissue and bone, which regulate each other through feedback mechanisms (14).

In the present review, we will focus on the bone as an organ target of several signals from the adipose tissue, to answer the clinical question whether the bone of children and adolescents with obesity is unhealthy in comparison with normal-weight peers.

We will not address the role of the bone in the regulation of physiological functions that are pivotal in the obesity status such as glucose homeostasis, energy metabolism, and appetite control. This side of the cross-talk has been brilliantly discussed elsewhere (15–18). In brief, the bone is the fourth glucose consuming organ after muscle, liver and adipose tissue, but unlike these tissues, it consumes glucose preferentially trough the aerobic glycolysis. Osteocalcin (OCN), a molecule secreted specifically by osteoblasts, regulates glucose metabolism, and browning of the adipose tissue in intertwined concert with insulin and leptin. OCN influences glucose homeostasis, modulating both insulin sensitivity, and secretion. Other osteokines participate to the fine control of energy metabolism. For instance, lipocalin2 (LCN2) regulates appetite by acting on the melanocortin 4 receptor pathway. Sclerostin, bone morphogenetic proteins 6 (BMP6) and 7 (BMP7) modulate browning of the adipose tissue.

The review of available data on the influence of bone on adipose tissue homeostasis is outside the scope of this report. It is our aim (i) to provide insights into the actions that adiposity exerts on bone accrual in children with obesity; (ii) to critically discuss the published clinical investigations on bone health status of children and adolescents with obesity; (iii) to argue for the beneficial impact of a healthy lifestyle on the child bone status, focusing on emerging dietary issues related to the intake of certain micro and macronutrients that characterize, for instance, the Mediterranean Diet (MD), i.e., polyunsaturated fatty acids (PUFAs) and polyphenols.

## Bone Development, Growth, and Puberty

The human skeleton undergoes several changes in size and shape during the different stages of life. Childhood and adolescence are characterized by rapid and significant longitudinal bone growth, areal bone expansion, and bone mineral accrual (19). Ninety percent of the peak bone mass is achieved in late teenage and the amount of bone mass reached by this age predicts BMD in adulthood (20, 21) (see glossary in Table 1).

**Figure 1:**
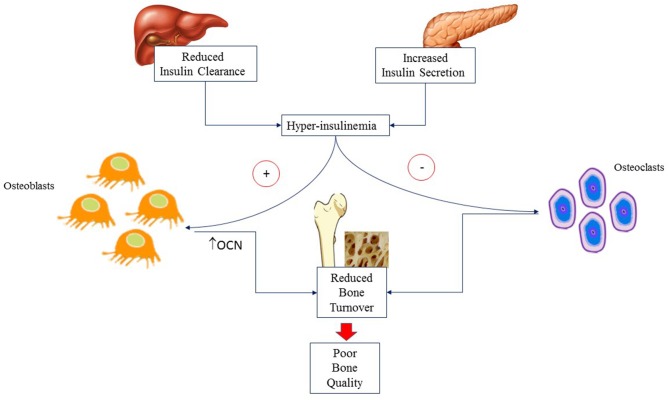
Hyperinsulinemia is due to altered insulin secretion and clearance that are commonly associated with obesity. It acts on osteoblasts causing reduced bone formation but also decreased number of osteoclasts and markers of bone resorption. The final result is reduced bone turnover and hence poor bone quality. OCN, Osteocalcin.

Genetics accounts for 60–80% of difference in BMD during teenage (22). Hormonal milieu (23), individual and environmental modifiable factors (i.e., body weight, adiposity, diet and PA) influence BMD later in life (24). Bone mass is progressively accrued from birth through childhood. At puberty, there is an impressive acceleration of BMD accrual secondary to the influence of anabolic hormones such as growth hormone (GH), insulin like growth factor 1 (IGF-1) and insulin. GH and IGF-1 promote osteoblast differentiation, myogenesis and muscle development (25, 26). Insulin enhances osteoblast development, promotes OCN expression (27) and reduces bone reabsorption. At puberty, insulin secretion increases physiologically but the rise is higher in adolescents with obesity who develop mild to severe hyperinsulinemia. In a mice model of reduced hepatic insulin clearance, hyperinsulinemia has been associated with higher trabecular and cortical BMC, reduced bone formation but also decreased number of osteoclasts and markers of bone resorption. These findings suggest that hyperinsulinemia is associated with reduced bone turnover and, consequently, poor bone quality as shown schematically in Figure 1 (28).

During puberty, boys present increased bone width and size as compared with girls of the same age due to the action of testosterone on periosteal apposition (29). There is no significant difference between sexes in cortical thickness (30). Estrogens influence bone development in both sexes causing increased osteoblast differentiation and reduced osteoclast lifespan. Estrogen deficiency leads to osteoblast apoptosis, oxidative stress, increased osteoblast nuclear factor kappa-light-chain-enhancer of activated B cells (NF-κB) activity and high level of RANKL (receptor activator of NFκB ligand)/osteoprotegerin (OPG) ratio resulting in enhanced bone resorption (31). The maximal gain in bone mass is seen ~6 months after the adolescence growth spurt. Nevertheless, bone mass, and density continue to increase over years thereafter (29).

Among the modifiable factors of an individual affecting BMD, excess weight and adiposity are major players for bone accrual. Studies have reported a positive effect of fat mass on BMD in prepubertal children (6) and adults (32, 33). However, it seems that the hormonal milieu of puberty inverts the positive trend of the association between adiposity and BMD that, indeed, becomes negative after puberty (34–37).

## Bone Marrow Development and Adipose Tissue

Bone marrow (BM) consists primarily of adipocytes (yellow marrow areas) or adipocytes and hematopoietic red blood cells (red marrow areas), that fill the cavities within the trabecular bone. Hematopoietic stem cells (HSCs) are hosted in BM microenvironments or niches where a variety of cells and molecules exert a fine-tuned regulation of their survival, self-renewal, differentiation and retention (38).

BM adipocytes arise from the differentiation of mesenchymal stem cell (MSCs). They belong to a heterogeneous population whose metabolism, lipid composition, secretory capacities and functional responses depend on their location within the BM. At birth, the BM is fully hematopoietic and contains no adipocytes. Soon after birth, adipocytes start to differentiate from MSCs. The hematopoietic BM converts gradually to fatty marrow. This conversion takes place initially from distal toward central skeleton and continues throughout aging (39). In long bones, tissue replacement starts in diaphysis. BMAT replacement occurs at the age of 10 years-old in the femur diaphysis, and at the age of 30 years in the distal metaphysis (40). By the end of adolescence, hematopoietic marrow remains in the proximal metaphysis of femur and humerus, spine, sternum, ribs, and skull. By early adulthood BM adipocytes that have been developing in the prenatal skeleton and increasing in number with aging, occupy up to 70% of the BM microenvironment (41).

**Figure 2:**
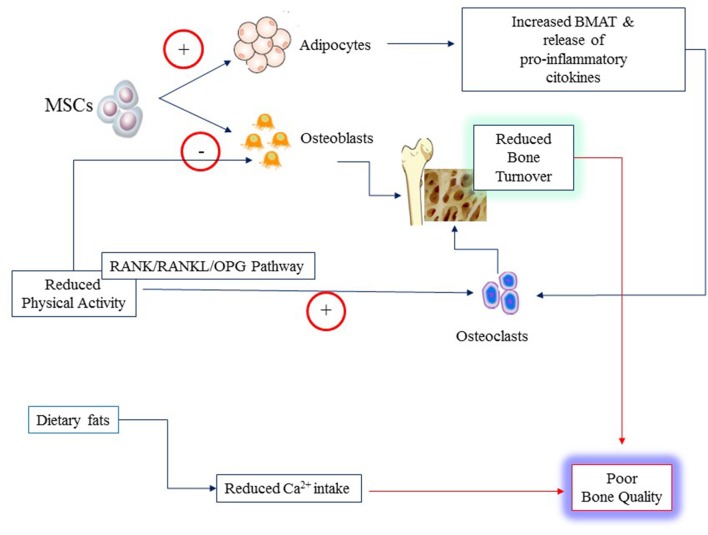
Poor bone quality in young individuals with obesity may result from several mechanisms: increased rate of differentiation of Mesenchymal Stem Cells (MSCs) to adipocytes at the expense of osteoblasts; reduced physical activity that alter the balance between osteoblast and osteoclast activities through the RANK/RANKL and osteoprogerin (OPG) pathways; reduced calcium (Ca^2+)^ availability from the diet owing to the high content of fats.

Obesity influences bone microenvironment and health by different mechanisms that are concisely depicted in Figure 2. Firstly, obesity diverts MSC differentiation toward the adipocyte line at the expense of osteoblasts. This imbalance results in reduced bone formation and increased BMAT (10). Secondly, obesity that is often characterized by reduced levels of physical activity (PA), promotes osteoclast activity and bone resorption triggering the expression of receptor activator of nuclear factor κ B (RANK), favoring binding to its ligand RANKL, and conversely inhibiting OPG pathway (11). OPG is the decoy receptor that binds and thereby opposes RANKL (42). RANK is expressed on monocytes and macrophages. Binding of RANKL causes fusion of monocytes and macrophages and their differentiation into osteoclasts ultimately leading to increased bone resorption (43). Most inflammatory adipokines (i.e., tumor necrosis factor-α, TNF-alpha; interleukins (IL, IL1, IL6, IL17) upregulate RANK/RANKL expression (11). Thirdly, obese children have often a diet particularly rich in fats that may reduce intestinal calcium absorption causing decreased calcium availability for bone formation and boosting further inflammation (44–46). Fourthly, physical activity is a major mechanical stimulus for bone accretion and is often reduced in obese children.

On the other hand, obesity, intended as excess adiposity in the visceral and subcutaneous compartments, increases the mechanical load to the bone and, by doing so, promotes accrual of the cortical bone that represents a kind of armor for the whole body. Cortical bone is ~80% of the total bone mass and is crucial in weight-bearing and physical performance (47). Computed tomography (CT) studies (48) demonstrated an inverse relationship between BMAT and BMC in the axial and appendicular skeleton of healthy adolescents and young adults regardless of sex. Such relationship was unrelated to whole body adiposity. The excess BMAT may reduce BMC by replacing bone cells with adipocytes thus altering the bone micro-environmental milieu and microstructure. Conversely, generalized adiposity with increased depots of subcutaneous and visceral tissue may favor bone accrual by exerting a mechanical stimulus on the bone.

## Excess BMAT and Inflammatory Cytokines

In children and adolescents, the ectopic deposition of BMAT in skeleton sites where it is expected to develop later in life, might cause an imbalance between osteoblastic and osteoclastic activities, leading to reduced turnover, bone fragility (49) and early-onset osteoporosis. Indeed, higher fraction of BMAT was associated with lower BMD values (50). A magnetic resonance imagining (MRI) study of 185 healthy children (5–18 years old) with wide range of body mass index (BMI) found an inverse correlation between BMAT and right femoral cortical bone area after adjusting for weight, total body fat, subcutaneous adipose tissue, visceral adipose tissue, and skeletal muscle (51). However, a longitudinal study demonstrating early-onset osteoporosis in individuals with obesity is lacking.

Excess BMAT releases a number of pro-inflammatory molecules. Most of them trigger RANK pathway (11), up-regulate osteoclast formation and activation (11, 52) and exert paracrine effects that vary in the different skeletal compartments (i.e., trabecular vs. cortical) (48, 53, 54) and sites (i.e., weight-bearing vs. not-weight-bearing). The adipose tissue expresses many adipokines, i.e., leptin, adiponectin, IL6, IL10, monocyte chemotactic protein-1 (MCP-1), TNF-α, macrophage colony stimulating factor. The obesity status is also characterized by altered levels of molecules that either affect bone growth (GH, parathyroid hormone, angiotensin II, 5-hydroxy-tryptamine) or modulate signaling pathways within the bone and the muscle tissues (i.e., advanced glycation end-products, myostatin and irisin). The effects of these molecules on preferential differentiation of MSCs to adipocytes or osteoblasts and bone remodeling have been investigated in cellular and animal models. The results of these studies are not entirely consistent, due both to heterogeneity of experimental designs and complex nature of adipokine signaling. Pro-inflammatory adipokines down-regulate osteoblasts, osteocytes, and muscle cells and upregulate osteoclasts.

The effect of some adipokines is dose-dependent. At physiological concentrations leptin promotes the production of the osteoclastogenic inhibitor OPG by osteoblasts but at higher concentrations (i.e., 10-fold higher than normal) is associated with inhibition of OPG and production of RANKL by osteoblasts (55). Another peripheral mechanism of leptin action at physiological concentrations is the modulation of MSCs differentiation to osteoblasts and their bone mineralization. In a murine model of conditional regulated leptin receptor gene recombination, it was found that leptin helps maintain MSCs in an undifferentiated state and promotes mineralization of more differentiated osteoblasts (56). In a mouse model of high-fat diet, increased levels of circulating leptin were associated with reduced tibial trabecular thickness, volume, and density (57), whereas mice treated with a leptin antagonist showed increased lumbar trabecular thickness (58). In general, leptin deficiency in mice affect cortical bone leading to reduced thickness (59). Leptin deficient mice had high bone mass that was reverted by cerebral intraventricular injection of leptin with consequent loss of trabecular bone. In tail suspended rats, lower doses of leptin were protective of bone loss, while high doses caused increased bone resorption and reduced bone formation (60).

Finally, leptin acts through a central neuroendocrine pathway that seems to involve hypothalamus, brainstem, and raphe nuclei likely via serotoninergic neurons to negatively regulate bone mass (61).

Overall these findings indicate that the net *in vivo* effect of leptin on the human bone physiology results from the balance of very complex interactions (62).

In obese children, Dimitri et al. found a negative association between leptin and BMC (63) and between its unbound form and OPG (64, 65). Leptin concentrations were inversely related to radial cortical porosity, radial cortical pore diameters, and tibial trabecular thickness (66).

Evidence from experimental models on the effect of the major adipokines on bone health is reported in Table 2.

**Table 2:** Adipokines and adipose tissue derived molecules that affect bone health.

**Molecule**	**Effect on bone**	**Reduced or increased release in obesity**	**Possible impact on bone health in obesity**
Adiponectin	Enhances MSC differentiation to osteoblasts (67, 68)	Reduced	Prevailing bone resorption
	Suppresses osteoclasts activity (68)		
	Enhances osteogenesis throughout the Wnt/β catenin pathway (67)		
AGEs	Inhibits osteoblast growth (69); Induces osteocyte apoptosis (69) causing cortical bone deterioration (70)	Normal/increased	Prevailing bone resorption
	Inhibits osteoblastic differentiation of stromal cells by decreasing osterix expression and partly by increasing RAGE expression.		Reduced bone accrual
Chemerin	Up-regulates osteoclast differentiation of HSCs (71)	Increased	Prevailing bone resorption
	Enhances adipocytes differentiation from MSCs (72)		
IL-6	L-6 acts directly on marrow-derived osteoclasts to stimulate release of “osteotransmitters” that act through the cortical osteocyte network to stimulate bone formation on the periosteum (73)	Increased	Altered bone structure
Leptin	At physiological concentration, promotes osteoblast proliferation and differentiation (63)	Normal	Prevailing bone resorption
	At supra-physiological concentration, favors bone resorption by increasing RANKL expression (64)	Increased	
	At physiological concentration, inhibits adipocyte differentiation and osteoclastogenesis through generation of osteoprotegerin (74).	Normal	Prevailing bone accrual
	At supra-physiological concentration, inhibits osteoblast proliferation via circadian clock genes (75, 76).	Increased	Reduced bone accrual
LPN-2	Interferes with osteoblast differentiation causing reduced bone accrual and turnover and enhances bone resorption in mice overexpressing Lcn2 (77) but there is	Increased	Unclear
	Reduced osteoblast number and bone formation rate in Lcn2^−/−^ mice (78)		
MCP1	Promotes osteoclastogenesis binding CCR2 and triggering JAK/STAT and Ras/MAPK signaling pathways (79)	Increased	Prevailing bone reabsorption
	Triggers RANK-pathways (80)		
TGFβ	Induces osteoblast-specific gene expression (81, 82)	Increased	Bone accrual
	Causes perilacunar/canalicular remodeling (83)		Poor bone quality
TNF-α	Promotes osteoclast formation and bone resorption *in vivo* synergistically with RANKL and IL6 (84)	Increased	Prevailing bone reabsorption
	Inhibits osteoblastogenesis and enhances adipocyte differentiation (85)		Prevailing bone reabsorption
	Induces recruitment and differentiation of peri-fracture mesenchymal stem cells favoring bone healing (86, 87).		Bone healing
	Reduces osteoblasts viability and induces apoptosis in cell model of hyperglycemia (88)		Reduced bone accrual
Visfatin	Increases matrix mineralization and reduces collagen type I expression. Interferes with MSC differentiation (89)	Increased	Bone fragility/reduced bone accrual

AGEs, Advanced glycation-end products; HSC, Hematopoietic Stem cell; IL-6, Interleukin-6; LPN2, Lipocalin 2, MSC, Mesenchymal Stem Cell; MCP1, Monocyte Chemotactic Protein-1.

*TGF-β, Transforming growth factor β; TNF-α, Tumor Necrosis Factor-α; RAGE, receptors for advanced glycation end-products; RANKL, receptor activator of NFκB ligand; CCR2, C-C Motif Chemokine Receptor 2*.

In addition, some inflammatory molecules that are not directly expressed by adipocytes but by the immune cells embedded within the BMAT are also able to divert MSCs toward adipocytes at expense of osteoblasts. This is the case of some members of the TNF-α superfamily such as TRAIL and LIGHT/TNFSF14. The former has pro-osteoclastogenic and osteoblastic pro-apoptotic effects, whereas the latter has a well-defined pro-osteoclastogenic effect in different models of bone disease. In a pilot investigation, Brunetti et al. found higher serum levels and monocyte expression of LIGHT in 10 children with obesity as compared to healthy controls. LIGHT serum levels were inversely correlated with the bone transmission time Z-score measured by quantitative ultrasounds (52).

## Exercise and Mechanical Signals Are Anabolic to Skeletal Tissue

Sedentary lifestyle influences negatively bone health reducing mass accrual and promoting tissue absorption. MSCs and their lineage-differentiated progeny are mechano-sensitive. Exercise induces the transmission of mechanical signals across the plasma membrane of MSCs through cytoskeletal proteins and transmembrane-bound integrins into the nucleus. Mechanical stimuli activate signaling cascades and induce cytoskeletal adaptations that initiate osteogenic, chondrogenic and myogenic differentiation and inhibit adipocyte differentiation down-regulating the peroxisome proliferator-activated receptor gamma (PPARγ)-driven adipogenic pathways. Bed stay and bone disuse increase the expression of PPARγ in MSCs and RANKL in BM, which promote osteoclast mediated bone resorption. The effects of bone disuse are promptly reverted by PA and mechanical stimuli [recently revised in (90)].

In a meta-analysis of 27 studies investigating the effect of PA on bone development and accrual, Berhinger found that weight bearing and exercise promote BMC gain. This effect is more evident during pubertal growth than after puberty, suggesting that the skeleton is more responsive to PA during this age window. Nevertheless, they concluded that there is no apparent correlation between PA intensity and BMC (91).

It is reasonable that weight-bearing exercise, the cornerstone in treatment and prevention of adult osteoporosis, works also in children and adolescents with obesity to prevent fragility and to improve bone health. Intramuscular fat impairs muscle strength and PA counteracts the accumulation of fat in the muscle fibers. Therefore, PA produces a range of mechanical stimuli and soluble signals across the skeleton and musculature targeting cells, tissues and organs that result in increased lean muscle mass, BMC, turnover rate and reduced low-grade local and systemic inflammation. In the absence of mechanical load, osteoclast-mediated resorption is accelerated by the secretion of inflammatory adipokines released into BM, i.e., the transforming growth factor-β (TGFβ). High levels of inflammatory adipokines result in bone resorption that is not accompanied by bone formation. It is noteworthy that increased levels of TGFβ cause impaired calcium gradient across muscle fibers and ultimately affect bone-muscle crosstalk (90, 92).

PA stimulates muscle release of irisin, a hormone-like myokine. Once released into the circulation, irisin acts on white adipocytes to induce their browning response but it may be also beneficial for bone health. In mice, irisin produced during exercise had positive effects on cortical mineral density and geometry (93, 94). In 6–8 years-old children, Soininen et al. found a significant positive association between circulating irisin and BMD (95).

The adequate intake of calcium and vitamin D seems to play a key role in bone mass accrual at puberty enhancing the beneficial effects of exercise. A recent systematic review has shown an additive effect of calcium intake and PA on bone health in children with low dietary calcium intake. The effect was more evident when the supplementation was done in early puberty and in weight-bearing bones (96). Nevertheless, the available data are still inconclusive as based on small sample sizes, no randomized controlled trials and no longitudinal studies.

## Techniques to Investigate Bone Density, Content, and Microstructure

Bone mineral accrual is estimated in clinical practice by Dual X-ray absorptiometry (DXA). DXA scan generates a 2-dimensional (2D) planar image and provides a measure of BMC (g) and areal-BMD (aBMD, g/cm^2^) (Table 1). Given a reference population, the DXA software generates an age and gender specific z-score (standard deviation score) (97). The technique is not free of limitations. Data produced by equipment of different brands (Hologic vs. Lunar) have their own software and reference population data which do not allow consistent comparisons. Reference standards need to be made in full awareness of sample size, gender, age, and ethnicity of the reference population (98). The most serious limitation of DXA is that it provides a 2D image of a 3D structure. Thus, aBMD overestimates true bone density (g/cm^3^) in taller children with larger bones while underestimating it in shorter children with smaller bones. Obese children are often taller and tend to have larger bones. In these children, the accuracy of DXA measurements is reduced and clinical interpretation biased (99, 100). Indeed, interpretation of DXA parameters of an obese child remains challenging. Bone density may be corrected for body size to overcome this limit, but a consensus on this procedure is yet to be reached (97, 98, 101–104). It is a matter of fact that obese children have lower BMC when it is corrected for body size (101, 102, 105). Current guidelines recommend the use of bone mineral apparent density (BMAD) (Table 1) for the assessment of lumbar bone density and height for age adjusted Z-score for the assessment of total body less head (TBLH) bone density (106). The adjustment of BMC, bone area and aBMD for soft tissue (muscle vs. adipose tissue) may represent another option to test (102).

Progress in bone imaging has shifted attention to the bone microarchitecture as proxy of bone quality and health. Peripheral quantitative CT (pQCT) scanning and high-resolution pQCT (HRpQCT), with spatial resolution of 64 mm, allow measuring *in vivo* the volumetric parameters of trabecular and cortical compartments. They provide a sort of virtual “bone biopsy” that investigates bone microarchitecture. In long bones, pQCT reports global bone strength, expressed as thickness, periosteal and endosteal circumference and section modulus (102). The application of micro-fine element analysis (FEA), an engineering tool to derive bone mechanical properties as they relate to its microstructure, to HRpQCT images of ultradistal tibia and radius provides an estimate of bone strength relative to load and stiffness (107). Cross sectional area of lower limb and muscle area can be derived from this technique as indicators of body composition (102, 108) thus providing information on the “muscle-bone” unit.

MRI makes a 3D reliable reproduction of trabecular and cortical bone, appendicular and central skeleton in different anatomical planes, with the advantage of not exposing the child to ionizing radiations. However, its use is currently limited to research purposes (51, 109).

Both HRpQCT and MRI techniques may be particularly useful for assessing bone quality during puberty when bone microarchitecture undergoes important changes.

Quantitative ultrasound (QUS) is a radiation-free technique that informs about the mineral status measuring the amplitude-dependent speed of sound (Ad-SoS; m/sec), Broadband Ultrasound Attenuation (BUA; dB/MHz), and the bone transmission time (BTT) in tibia and radius. Unfortunately, there are no reference values for pediatric population and for different ethnicities (110). The International Society for Clinical Densitometry recommends its use for osteoporosis management in adults (111).

Strength and limitations of the imaging techniques are summarized in Table 3.

**Table 3:** Strengths and limitations of the imaging techniques.

**Imaging Techniques**	**Advantages**	**Disadvantages**
DXA	Gold standard, widely known and used Short analysis time Good accuracy	Use of radiation (albeit in small doses: 6.7–31 μSv) Need for stillness and compliance Dual dimension images Lack of solid pediatric reference curves Medium cost
HR-pQCT	Measure of cortical and trabecular volumetric BMD Low dose of radiation (<2 μSv) Measure of bone microarchitecture Portable and less expensive machine	Difficult child positioning High dose of radiation (although in limited area) Not clinically available Lack of reference values (RV) for clinical routine use
MRI	Measure of cortical and trabecular volumetric BMD No radiation Measure of microarchitecture	Difficult child positioning Long scan time High potential for motion artifact Lack of accessibility Lack of RV High cost
QUS	Measure of bone mineral status by computing Ad-SoS; BUA and BTT No radiation	Difficult use in pediatric age Lack of reference value in pediatric age No indication for clinical routine to date

*DXA, dual X-ray absorptiometry; HrpQCT, high resolution pQCT; MRI, magnetic resonance imagining; QUS, quantitative ultrasound*.

## Risk of Extremity Fractures in Obese Children

Extremity fractures are very frequent events in children and adolescents regardless of their body weight (112) with incidence peaking between ages 11–14 years in boys and 8–11 years in girls (113). There are raising concerns about a negative impact of fatness on bone mass since children with obesity are overrepresented in fracture groups (114–120), especially fractures of upper and lower extremities (118, 119, 121). A systematic review of 6 articles (*N* = 4,594 children; 867 with obesity and 3,727 normal-weight children) concluded that children with obesity have 25% higher risk of extremity fractures than normal-weight peers and also higher mortality rate (4.7 vs. 2.8%, respectively). As the authors acknowledged, confounders (ages, different mechanisms of injury, sample size, comorbidities) were not considered in the analysis that included exclusively retrospective studies of children involved in traumatic injuries (121). Nonetheless, a population-based study of 913,178 children aged 2–19 years-old confirmed the increased risk. The study, which did not provide information on mortality and therefore was not included in the former systematic review, found increased odds ratio of lower limb fractures in children with obesity. The risk increased with body weight. Children with extreme obesity had around 50% increased risk of foot, ankle, knee and leg fractures (9).

On the other hand, the increased risk of fractures might be due to weight-related clumsiness, postural instability and impaired gait that make children with obesity prone to fall and experience fractures (122–126).

## BMC and BMD in Children With Obesity

There are inconsistencies between studies that investigated BMC/BMD and their relationship with fat mass and those investigating bone fragility and risk of fractures in children with obesity.

Both a history of prior fracture and overweight were predictors of future fracture in a cohort of 200 white girls (aged 3–15 years, half with recent forearm fracture and half age-matched with no history of bone fractures) followed up for 4 years. More than one third of girls with history of fracture had reduced lumbar BMD. An unexpected proportion of them was overweight (105). Likewise, in boys aged 3–19 years-old, excessive weight was a risk factor for distal forearm fractures. Low BMC, aBMD, BMAD and high fat mass were associated with increased risk of distal forearm fracture in boys. More than one third of the cases had reduced lumbar density and BMAD at radius, hip, and spine (117).

The same research group confirmed results in a different sample of 90 children with history of at least two forearm fractures in life. Children were scanned at different bone sites. Regions of interest were ultradistal radius, one-third radius, neck of femur, hip trochanter, lumbar, and total body. Early age of first fracture and overweight (33.3 vs. 15.5%) were both associated with enhanced risk of repeated fractures. As the number of fracture episodes increased, both BMC and BMD z-scores decreased, in particular at the ultradistal radius. Overweight children had the lowest mean BMC Z score at the ultradistal radius [−0.66 (1.22)] that was inversely associated with BMI z-score (115).

In the 8,348 young (8–18 years-old) participants to the US National Health and Nutrition Examination Survey (NHANES), BMD was adjusted for lean body mass, fat mass, ethnicity, age and gender. After adjustment for total body fat mass and percent body fat, children with obesity showed reduced whole body aBMD and lumbar spine aBMD, supporting the notion that people with obesity experience a real reduction of the bone mineral mass. In the study, pelvic aBMD was apparently not influenced by adiposity (127). These findings support an association between body weight or BMI and impaired BMC, at least at certain sites, with consequent increased risk of extremity fractures. Studies that focused on the association between adiposity, BMD and BMC, found that fat mass may have either a positive (5–7) or a neutral effect on bone (128, 129). A meta-analysis of 27 studies (*N* = 5,958 children) concluded (quality of evidence from moderate to high) that children with obesity have a significant higher bone mass than normal weight children as estimated by BMC, BMD, BMAD, and volumetric BMD (Table 1). Bone mass as estimated by whole body BMD was higher only in girls with obesity, but not in boys (7).

In a cohort of 3,082 children (mean age 9.9 years), Clark et al. found a positive association between whole body fat mass and TBLH bone area regardless of lean mass. In boys and prepubertal girls, fat mass predicted bone size gain after 2 years of follow up, independently of other factors suggesting that adipose tissue promotes bone growth in pre-pubertal children (6).

## Microarchitecture and Bone Strength by QCT in Children With Obesity

Likewise, the results from pQCT/HR-pQCT investigations in children with obesity are controversial. 3D-cortical and trabecular microstructure and biomechanics at load-bearing and no-load bearing sites were studied in obese and lean children. Cortical porosity and mean cortical pore diameter at the radius were reduced in the obese group. Children with obesity showed reduced tibial trabecular thickness, increased trabecular numbers at the distal tibia but no difference in biomechanical properties of the bone (66). In a previous study, Farr et al. demonstrated no differences in the cortical and trabecular bone microarchitecture of obese and lean subjects (130).

QCT provides also the bone strength index (BSI, mg/mm^4^) that is an estimate of strength in response to compression at the distal end of long bones and is calculated as the product of total cross-sectional area (ToA) per total volumetric density (ToD)^2^. Overweight children had higher BSI than normal weight children at the tibia. Bone strength did not adapt to fat mass but to lean mass, suggesting that the bone strength, despite being increased, was not adequate to the larger fat mass of children with obesity (131).

The effect of adiposity on bone microstructure and strength may vary upon site of depot, i.e., fat mass seems to favor bone strength in lower limbs but not in upper limbs. Ducher et al. reported decreased bone strength at the forearm due to a greater percentage of fat mass in proportion to muscle mass (132).

In a cohort of 135 girls and 123 boys (age 8 years-old), bone strength was measured in response to torsion at 11, 13, 15, and 17 years of age by DXA and pQCT. Girls with overweight at baseline had significantly greater bone strength than normal weight girls while boys with overweight at baseline had greater bone strength than normal-weight children only at tibia and femoral neck but not at radius. When strength was adjusted by biological age, it was reduced in both girls and boys with overweight in comparison with normal-weight peers. These differences were no longer present when adjusted for lean mass in girls suggesting that the association between adiposity and bone strength is gender-specific (133).

## Weight Loss and Physical Activity

In adults, weight loss leads to the reduction of BMD and enhances the risk of fractures (134). The effect of weight loss on the adolescent's bone health is still matter of debate.

In 92 obese adolescents engaged in 12 months weight loss trial, whole-body and vertebral BMC did not change respect to baseline despite the weight loss. On the contrary, upper and lower limb-specific BMCs for height were reduced, while lumbar-specific BMC for height was increased as compared to controls who were 54 normal weight and 12 overweight age- and height-matched adolescents (135). Gajewska et al. evaluated BMD, BMC and circulating adipokines in 40 pre-pubertal obese children before and after 1 year of weight loss program. They observed a positive association between the amount of weight reduction and the increase of BMC and BMD as absolute values. When the BMD z-score was considered, they found a reduction in BMD z-score that was positively correlated with the BMI z-score, suggesting a deceleration in bone mass gain in parallel with the weight loss. BMD z-score reduction was associated with decreased levels of bone alkaline phosphatase, leptin and sclerostin that are all markers of bone accrual, while levels of adiponectin increased (37).

Aerobic and anaerobic PA favor loss of weight, visceral and subcutaneous adiposity, and ameliorates insulin sensitivity in subjects with obesity. The combination of aerobic and non-aerobic activity (resistance training) improved BMC. Children who underwent a resistance training program showed higher BMC than peers under aerobic training alone but no difference in BMD (136). Conversely, sedentary habits were associated with decreased BMC (137).

Combination of aerobic plus resistance trainings was associated with improvement in lean body mass, higher levels of adiponectin, and reduced circulating leptin and low grade of inflammation in children and adolescents with obesity (136).

In a recent study, Munoz-Hernandez et al. investigated the combined effects of the Mediterranean diet and PA in overweight/obese children aged 8–12 years old. While they found no evident effect of the diet on bone health, a moderate to vigorous intensity PA was associated with the increase of both BMC and BMD. In particular, there was an increase of 10 g in BMC for 1 h/day increase of PA and 48 min/day reduction of sedentary habits (138).

One of the mechanisms of PA related improvement of bone health (93, 139) is the enhanced release of irisin. In a small sample of healthy children (aged 7–13 years), with a comparable intensity level of PA, circulating levels of irisin were positively associated with bone mineral status, assessed by QUS (140). Irisin contributes to browning of the adipose tissue, and in turn brown adipose tissue (BAT) seems to have a positive effect on cortical bone area. Indeed, Ponrartana et al. found a significant association between BAT volume and cortical bone area at the midshaft of the femur in 40 children and adolescents (141).

The evaluation of the net effect of weight loss on BMC and/or BMD is not straightforward for several reasons. Studies are heterogeneous in inclusion criteria, design, techniques of bone assessment, and data interpretation. Obese children and adolescents are taller than normal-weight subjects and have advanced bone-age and higher lean mass for height. Therefore, it is not easy to identify the appropriate controls. Pubertal spurt and rate of growth are important confounders. Differences in diet habits, micro to macronutrients balance, and intake of micronutrients are also important confounders. In addition, a weight loss program is often part of a lifestyle intervention that includes PA. Vigorous PA influences bone health (142), especially during growth, contributing to the bone mass peak, femoral neck BMD, and bone strength (142–146). Finally, small-size samples are a limitation for all these studies (147–149).

Regular aerobic and resistance (weight-bearing exercise) PA must be encouraged in young people with obesity representing an effective strategy to sustain bone tissue while ameliorating metabolic obesity-related morbidities (162–164).

## Emerging Dietary Issues: Polyunsaturated Fatty Acids and Polyphenols

Adequate and balanced dietary intake of micro and macronutrients contributes to bone health. Mediterannean diet ensures the beneficial intake of micro and macronutrients, including valuable lipids (165–172).

Children with obesity have a diet often poor in micronutrients (i.e., calcium, vitamin D etc.) and rich in saturated fatty acids (SFAs) that cause decreased calcium availability for bone formation and favor low-grade inflammation. Reduced levels of circulating 25-hydroxyvitamin D (25-OHD), which is pivotal for bone health and accrual, have been often reported in children with obesity as compared to normal weight peers. On purpose, we will not discuss this issue, which has been the focus of a number of recent excellent reviews (173–175). We will focus on the link between dietary fatty acids (FAs) and bone metabolism.

FAs provide an important energy source, participate in cell signaling cascades, and serve as essential mediators of inflammation. An adequate intake of FAs and the balance between pro-inflammatory and anti-inflammatory PUFAs is critical for the maintenance of cellular functions and tissue homeostasis. A diet rich in SFAs or with a high ratio of pro-inflammatory n-6 PUFAs (i.e., Arachidonic acid, ARA; and Linoleic acid, LA) to anti-inflammatory n-3 PUFAs (i.e., docosahexaenoic, DHA; and eicosapentaenoic, EPA) may lead to altered bone biology (176–186). The metabolism of osteoblasts relies on fatty acid β-oxidation for 40–80% (158) and osteoblasts express several intra- and extra-cellular FA receptors, including PPARs, G protein-coupled receptor 40/Free fatty acid receptor 1 (GPR40/FFAR1), GPR120, Toll-like receptor-4 (TLR4), cluster of differentiation 36/fatty acid translocase (CD36/FAT) and PUFA receptors, including GPR40 and GPR120, that all have an important effect on osteoblasts and bone (186). Inflammation is up-regulated by SFAs and n-6 PUFAs in osteoblasts, while mineralization and osteoblastogenesis are down-regulated. Excess of SFAs has lipotoxic effects on the osteoblasts, while n-3 PUFAs seem beneficial. The balance between n-6 and n-3 PUFAs appears to be a modulator of physiological osteoblastogenesis (150–152).

Likewise, osteoclasts express a number of FA receptors, such as GPR40, TLR4, CD36, PPARs and other scavenger receptors that are mainly involved in the transport and metabolism of cholesterol and estradiol into these cells. The role of SFAs and n-6 PUFAs in osteoclasts is unclear, and there is conflicting evidence as to whether they upregulate or downregulate osteoclastogenesis (161, 187). N-3 PUFAs showed inhibitory effects on osteoclast functions and were associated with increased BMD (155–159). As to the n-6 PUFA arachidonic acid, it was found to either up- or down-regulate osteclastogenesis (161, 187).

Molecular mechanisms exerted by FAs and PUFAs on osteoblasts and osteoclasts are reported in Table 4.

**Table 4:** Molecular mechanisms of FAs on osteoblasts and osteoclasts.

**Cell types**	**SFAs**	**Omega 3 PUFAs**	**Omega-6 PUFAS**
Osteoblasts	Increase inflammation (150) Decrease osteoblastogenesis Excess of SFAs have lipotoxic effects (151, 152)	Decrease inflammation (150) Promote osteoblastogenesis (151, 152)	Increase inflammation (150) Decrease osteoblastogenesis (151, 152)
Osteoclasts	Promote osteoclastogenesis? (153, 154)	Increase BMD (155–157) Inhibit bone resorption and osteoclastogenesis (158, 159)	Promote osteoclastogenesis? (159–161)

*BMD, bone mineral density; PUFAs, polyunsaturated fatty acids; SFAs, saturated fatty acids*.

The MD is beneficial for bone accrual and health also because of the high content in polyphenols. Polyphenols are phytochemicals normally found in fruits, plants and vegetables. They are protective against a number of chronic diseases, including osteoporosis. *In vitro* and animal models demonstrate that polyphenols can safeguard bone integrity by decreasing oxidative stress and inflammation, and modulating osteoblastogenesis/osteoclastogenesis balance as reviewed elsewhere in a recent issue of this Journal (188). Evidence in human studies, however, have provided no robust evidence on the beneficial effects of dietary polyphenols on bone mineral accrual and bone turnover markers in healthy adults and in patients with bone diseases (189). Furthermore, no clinical trial has been run in children with obesity to determine the effect of polyphenols on BMD.

A cornerstone in the diet is the intake of extra-virgin olive oil whose polyphenols can stimulate the proliferation of osteoblasts, modify their antigen profile, and promote alkaline phosphatase synthesis. Twenty-four hour treatment with 10^−6^ M of extra-virgin olive oil phenolic compounds (e.g., caeic acid, ferulic acid, coumaric acid, apigenin, and luteolin) modified gene expression of growth and differentiation/maturation osteoblasts markers such as the transforming growth factor b1 (TGF-1), TGF-b receptor 1, 2 and 3, BMP2, BMP7, run-related transcription factor 2 (RUNX-2), alkaline phosphatase (ALP), OCN, osterix Collagen type I (COL-I) and OPG (190). The effect of some phenolic compounds in the extra-virgin olive oil may be dose-dependent becoming toxic for cells at high doses cells (191). Indeed, treatment of mesenchymal cells with hydroxytyrosol 100 mM down regulated the expression of osteoblast differentiation markers and inhibited osteoblastogenesis (192), while the same dose of apigenin inhibited osteoblast differentiation markers (COL-I production, ALP, and calcium deposits) in murine osteoblasts (193).

Green tea is also rich of polyphenols (namely the epigallocatechin gallate) that improved bone health in a murine model of HFD induced obesity by the suppression of bone cell activity (194, 195). Vester et al. demonstrated that stimulation of primary human osteoblasts with low doses of green tea extracts during oxidative stress over 21 days improved mineralization and had beneficial effect on extra-cellular matrix production with higher gene expression of OCN and COL-I during osteoblasts differentiation (196).

A more recent study on peripheral blood mononuclear cells (PBMCs) of children with obesity and normal weight peers found that polyphenol cherry extracts have a beneficial effect on *in vitro* osteoclastogenesis determining a dose-dependent reduction of the expression of osteoclast genes, such as calcitonin receptor, cathepsin K and RANK. Twenty-four treatment of PBMCs from obese patients with 100 μg/ml polyphenol extracts from three different cultivars of cherries resulted in the significant reduction of the expression of TNF-a, whereas RANKL levels and cell viability were unchanged. Therefore, sweet cherry extracts, rich in anthocyanins, especially cyanidin-3O-rutinoside, and chlorogenic acids inhibited osteoclastogenesis *in vitro* mostly through a reduction of pro-osteoclastogenic cytokines (197).

## Conclusions

This review was narrative and highlighted the number of unsolved issues about the effect of excess adiposity on the developing bone. We believe that a systematic review was unfeasible owing to the small number of clinical studies available from the literature and, moreover, to their extreme heterogeneity in the design.

No *in vivo* study provided robust evidence that bone health is impaired in children with obesity enhancing the risk of extremity fractures. In these patients, fractures may occur more frequently due to clumsiness that enhances risk of falls and to excess weight that generates greater mechanical forces trough the extremity bones. On the other hand, some studies found increased bone mass in overweight children. However, technical issues, i.e., the lack of gold standard methodology to estimate BMC in these patients with increased body size, prevented us from suggesting conclusive results. Emerging imaging techniques will be likely helpful to solve the question of whether the bone health is impaired in relation to the increased size, greater fat and lean mass, taller height, and advanced bone age of the obese youth.

Experimental evidence in animal and cellular models demonstrates that replacement of BM by adipose tissue alters the bone microenvironment and promotes low-grade inflammation, which in turn result in reduced osteoblast and enhanced osteoclast activities. A rearrangement of the bone microarchitecture and a prevailing bone reabsorption seem to be the net results of these processes. Without any doubt, bone accrual in children with obesity is affected by different humoral stimuli, i.e., inflammatory cytokines, adipokines and myokines. Nevertheless, their bone is also subjected to a mechanical work-load that is beneficial for bone accrual and geometry. Finally, there are emerging dietary issues that strongly suggest that the adequate intake of some natural compounds is beneficial for bone health.

In conclusion, in answering the main question addressed in this review, i.e., whether the bone mineral content is higher in children with obesity as compared to normal-weight peers or the bone structure of these individuals is more fragile, larger population studies are needed that will consider not only fatness of participants but also their inflammatory status, lifestyle habits in terms of PA (weight-bearing vs. not wear-bearing), sedentary conducts and dietary intake of macro-and micronutrients.

## Author Contributions

DF analysis and interpretation of evidence and revision for important intellectual content. AA and MCo pubmed search and drafting of the manuscript. GU pubmed search and revision of the draft for important intellectual content. SC and MCa revision of the draft for important intellectual content. MM conception and design of the manuscript, analysis, and interpretation of evidence, drafting of the manuscript, and revision for important intellectual content.

## Conflict of Interest

The authors declare that the research was conducted in the absence of any commercial or financial relationships that could be construed as a potential conflict of interest.

## Abbreviations

Ad-SoS: amplitude-dependent speed of sound
AGEs: advanced glycation end-products
ARA: arachidonic acid
BM: bone marrow
BMAD: bone mineral apparent density
BMAT: bone marrow adipose tissue
BMC: bone mineral content
BMD: bone mineral density
BMI: body mass index
BMP6: bone morphogenetic protein 6
BMP7: bone morphogenetic protein 7
BSI: bone strength index
BTT: bone transmission time
BUA: broadband ultrasound attenuation
CCR2: C-C Motif Chemokine Receptor 2
CD36: cluster of differentiation 36
CT: computed tomography
DHA: docosahexaenoic acid
EPA: eicosapentaenoic acid
DXA: dual X-ray absorptiometry
FAs: fatty acids
FAT: fatty acid translocase
FEA: micro-fine element analysis
FFAR1: free fatty acid receptor 1
GH: growth hormone
GPR40: G protein-coupled receptor 40
GPR120: G protein-coupled receptor 120
HrpQCT: high resolution pQCT
HSCs: hematopoietic stem cells
IGF-1: insulin like growth factor 1
IL: interleukins
LA: Linoleic acid
LCN2: lipocalin2
MCP-1: monocyte chemotactic protein-1
MRI: magnetic resonance imagining
MSCs: mesenchymal stem cell
NF-κB: nuclear factor kappa-light-chain-enhancer of activated B cells
NHANES: National Health and Nutrition Examination Survey
OCN: Osteocalcin
OPG: osteoprotegerin
PA: physical activity
PPARγ: peroxisome proliferator-activated receptor gamma
pQCT: Peripheral quantitative CT
QUS: quantitative ultrasound
PUFAs: polyunsaturated fatty acids
RAGE: receptors for advanced glycation end-products
RANK: receptor activator of nuclear factor κ B
RANKL: receptor activator of NFκB ligand
SFAs: saturated fatty acids
TBLH: total body less head
TGFβ: transforming growth factor-β
TLR4: Toll-like receptor-4
TNF-alpha: tumor necrosis factor-α
WHO: World Health Organization.
